# Outcomes following laparoscopic versus open major hepatectomy for hepatocellular carcinoma in patients with cirrhosis: a propensity score-matched analysis

**DOI:** 10.1007/s00464-017-5727-2

**Published:** 2017-07-19

**Authors:** Hong-wei Xu, Fei Liu, Hong-yu Li, Yong-gang Wei, Bo Li

**Affiliations:** 1 Department of Liver Surgery, Center of Liver Transplantation, West China Hospital of Sichuan University, 37 Guo Xue Road, Chengdu, 610041 Sichuan China; 2 Department of Pancreatic Surgery, West China Hospital of Sichuan University, Chengdu, Sichuan China

**Keywords:** Laparoscopy, Major hepatectomy, Hepatocellular carcinoma, Liver cirrhosis

## Abstract

**Background:**

Laparoscopic major hepatectomy (LMH) for hepatocellular carcinoma (HCC) in patients with cirrhosis remains controversial due to limited reports in the literature. This study analyzed the perioperative and oncological outcomes of LMH for HCC with cirrhosis compared with open major hepatectomy (OMH).

**Methods:**

A retrospective analysis of patients with cirrhosis who underwent major hepatectomy for HCC between January 2015 and January 2017 was performed. Patients were divided into the LMH group and the OMH group. Short-term and oncological outcomes were compared before and after 1:1 propensity score matching (PSM).

**Results:**

A total of 103 HCC patients who received major liver resection were enrolled. There were 36 (35.0%) patients in the LMH group and 67 (65.0%) patients in the OMH group. After 1:1 PSM, well-matched 32 patients in each group were evaluated. Significant differences were observed in operative time (median, 255 vs. 200 min, *p* < 0.001) and Pringle time (median, 50 vs. 30 min, *p* < 0.001) between two groups. The blood loss and transfusion requirement were comparable in two groups. The rate of overall postoperative complications did not differ between two groups, while the incidence of ascites in the LMH group was significantly less than OMH group (9.4 vs. 31.3%, *p* = 0.030). The oncological outcomes between the two groups were comparable with regard to 2-year overall survival (85.7 vs. 86.7%, *p* = 0.694) and disease-free survival (72.9 vs. 81.5%, *p* = 0.990), respectively.

**Conclusions:**

LMH for HCC patients with liver cirrhosis showed comparable results in terms of postoperative morbidity and oncological outcomes compared with traditional open procedure. LMH may serve as a safe and feasible alternative for selected HCC patients with cirrhosis.

Hepatocellular carcinoma (HCC) is the third leading cause of cancer death worldwide [1]. Liver resection remains the mainstay of curative treatment for HCC due to the current donor shortage of liver transplantation [2]. Unfortunately, the majority of the HCC patients are suffered from liver cirrhosis, making hepatectomy more complex and risky in the setting of elevated portal pressure and impaired coagulation, particularly for patients requiring major hepatectomy [3–7].

As a less invasive surgical approach, laparoscopic liver resection (LLR) was first introduced in 1990s, and since then this technique has gained attention worldwide [8, 9]. In the statement by the First International Consensus Conference for Laparoscopic Liver Resection, laparoscopic left lateral segmentectomy was considered to be the gold standard approach with reported reduced blood loss, decreased rates of postoperative complications, and shorter hospital stays compared with traditional open liver resection [10, 11]. However, the progress of laparoscopic major hepatectomy (LMH) has been very slow worldwide because of its inherent technical difficulties and fear of uncontrollable bleeding during parenchyma dissection. By 2014, the Second International Consensus Conference for Laparoscopic Liver Resection was convened in Morioka, and laparoscopic minor hepatectomy was considered to be a standard surgical practice (IDEAL 3), while LMH was deemed only to be the “Exploration” stage as there is still risk associated with novelty (IDEAL 2b) [12, 13].

Over the past few years, advances in laparoscopic devices and experience have gradually expanded the indications for LLR and resulted in several centers reporting good outcomes after LMH [14–16]. What is more, for patient with liver cirrhosis, comparative studies have also proved the feasibility of this technique with good short- and long-term outcomes [3, 4, 7]. However, most of the published literatures focusing on cirrhotic patients were based on the laparoscopic minor resections, while the LMH for patients with cirrhosis have been performed only in a related small number of cases, making it remaining a debatable issue.

The aim of the present study is to analyze our initial experience with LLR and compare outcomes following purely LMH with open major hepatectomy (OMH) for HCC in patients with cirrhosis.

## Materials and methods

### Patient selection and evaluation

Data of all HCC patients who underwent major liver resection in West China Hospital (Sichuan University, Chengdu, Sichuan Province, China) between January 2015 and January 2017 were collected retrospectively from a prospectively established database. Patients following the inclusion criteria were selected: (1) male or female patients aged 18–75 years, (2) pathological confirmation of HCC with liver cirrhosis (stage 4 fibrosis according to the Metavir score) [17], (3) maximum tumor size ≤10.0 cm, (4) Child–Pugh class A liver function, (5) a 15-min indocyanine green (ICG) retention rate ≤10%, (6) without extrahepatic metastases, and (7) Eastern Cooperative Oncology Group (ECOG) score 0 or 1 [18]. The exclusion criteria were as follows: (1) pathological confirmation of mixed-type HCC with intrahepatic cholangiocarcinoma, simultaneous hemangioma, or traumatic liver rupture, (2) severe dysfunction of the heart, kidney, or other organs, and (3) history of any other malignancy.

Patients were divided into two groups as follows: the LMH group and the OMH group. Preoperative assessments included routine blood tests, liver function tests, serum alpha-fetal protein test, ICG clearance test, and 3-phase-enhanced computed tomography (CT), or magnetic resonance imaging (MRI) scans. Major hepatectomy was defined as the resection of three or more contiguous segments and the resection of posterior superior segments according to the second International Consensus Conference for Laparoscopic Liver Resection [12]. The primary endpoint of this study was overall survival (OS) and disease-free survival (DFS), while postoperative morbidity was the secondary endpoint. This study was approved by the institutional review board.

### Surgical procedure

**Fig. 1:**
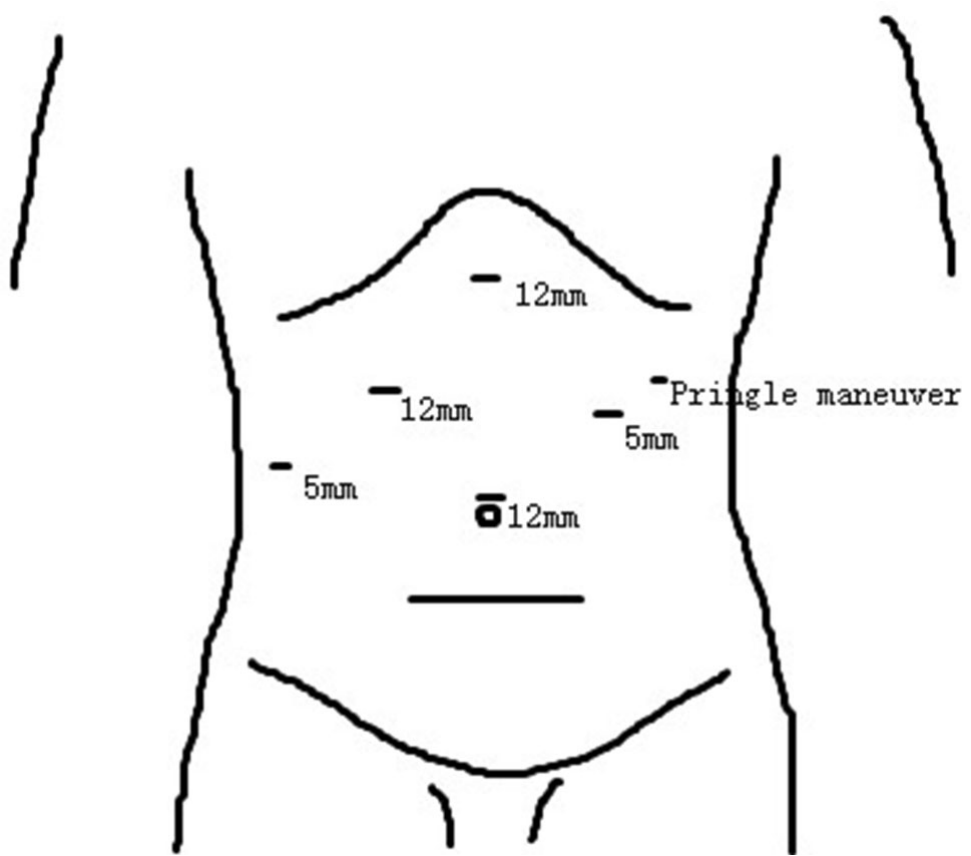
Port positions

**Fig. 2:**
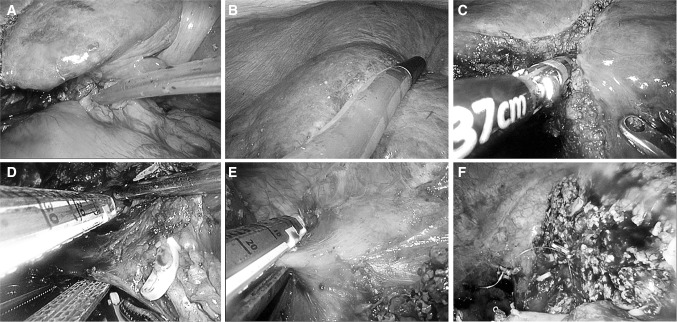
Surgical procedure (take right hepatectomy for example). **A** Pringle maneuver; **B** laparoscopic ultrasonography; **C** parenchyma dissection; **D** right portal pedicle transection; **E** right hepatic vein transection; **F** cut surface

For LMH, the detail of surgical approach has been previously described [19]. In brief, all patients underwent LMH were placed in left semi-decubitus position, followed by total intravenous general anesthesia. Carbon dioxide was insufflated to establish pneumoperitoneum, and the intra-abdominal pressure was maintained at 13 mmHg. Two 12-mm and two 5-mm trocars were usually used and adjusted according to the tumor location and extent of resection (Fig. 1). To control surgical blood loss, Pringle maneuver was routinely applied and the central venous pressure was maintained <5 mmHg (Fig. 2A). Laparoscopic ultrasonography was performed to confirm lesion position and guide resection (Fig. 2B). The superficial parenchyma was dissected by harmonic scalpel (Ethicon Endo-Surgery, USA), while the deeper tissue was dissected by laparoscopic cavitron ultrasonic surgical aspirator (CUSA, Valleylab, Inc, USA) or LigaSure (ValleyLab, Inc, USA) (Fig. 2C). For vessels ≥5 mm in diameter, Hem-o-lock clips (Weck Surgical Instruments, USA) or Titanium clips were used to achieve vascular control. The hepatic veins and portal pedicles were transected by a laparoscopic linear stapler (Fig. 2D, E). The specimen was then placed into a plastic bags and extracted through an enlarged port in the upper abdomen or the suprapubic transverse incision. Abdominal drainage was routinely placed on the cut surface (Fig. 2F).

For OMH, all patients under the same anesthesia were placed in supine position and the laparotomy was performed through an reverse L-incision. Cavitron ultrasonic surgical aspirator or clamp crushing was used as the main methods for parenchyma transection. Pringle maneuver and bipolar electrocoagulation were usually applied to control blood loss.

### Postoperative hospitalization and follow-up

Liver function tests and routine blood tests were conducted on postoperative days 1, 3, 5, and 7. An ultrasound imaging was usually performed in all patients before discharge. Clavien classification was used to grade postoperative complications and a major complication was defined as Clavien–Dindo ≥3 [20]. Liver-specific complications were categorized as follows: liver failure was identified in terms of the “50–50 criteria” [21]; ascites was defined in which the postoperative daily abdominal drainage exceeding 10 mL/kg of body weight [22]; hemorrhage was defined as a drop in hemoglobin level >3 g/dL postoperatively compared with the postoperative baseline level and/or any postoperative transfusion of packed red blood cells for a falling hemoglobin [23]; biliary leakage was defined by a bilirubin concentration in the drainage fluid at least three times than that in serum concentration on or after postoperative days 3 [24]. Postoperative mortality were defined as those occurring within 90 days of surgery.

After discharge, all patients were followed every 1–3 months during the first year and every 3–6 months afterwards. Routine blood tests, liver function tests, tumor maker tests as well as abdominal ultrasonography were usually measured at each follow-up. Three-phase-enhanced computed tomography or magnetic resonance imaging was performed once the recurrence was suspected in ultrasound imaging.

### Statistical analysis

To limit the selection bias arising from lack of randomization, 1:1 propensity score matching (PSM) was performed between the LMH and OMH groups using the nearest neighbor matching method based on the following variables: age, sex, body mass index, American Society of Anesthesiologists grade, preoperative blood test, previous abdominal surgery history, comorbidities, tumor size, and tumor location [25]. Baseline continuous variables between the two groups were analyzed using Mann–Whitney *U* test before PSM, while the Wilcoxon signed-rank test was performed after PSM. Categorical variables were analyzed using Chi-square test and McNemar’s test before and after PSM. Survival was assessed using the Kaplan–Meier method and compared based on the log-rank test. Probability (*p*) values <0.05 were considered statistically significant. All analyses were performed with SPSS version 22.0 (IBM SPSS Inc, Chicago, IL).

## Results

### Baseline characteristics

A total of 336 HCC patients who received major liver resection between January 2015 and January 2017 were retrospectively analyzed. Of which, 103 patients meeting the inclusion and exclusion criteria were selected for comparison (233 patients were excluded for reasons including that not stage four fibrosis, *n* = 145; tumor size >10 cm, *n* = 87; mixed-type HCC, *n* = 1, respectively). There were 36 (35.0%) patients in the LMH group and 67 (65.0%) patients in the OMH group. The baseline patients’ demographics before PSM showed significant differences in terms of ALB (median, 42.3 vs. 40.6 g/L, *p* = 0.027), PLT (median, 127.0 vs. 99.0 10^9^/L, *p* = 0.013), and tumor size (median, 4.3 vs. 6.0 cm, *p* = 0.036), respectively. After 1:1 PSM, there were 32 patients in each group with comparable baseline characteristics (Table 1).

**Table 1:** Baseline characteristics before and after propensity score matching

Variables	Before matching			After matching		
	LMH (*N* = 36)	OMH (*N* = 67)	*p* value	LMH (*N* = 32)	OMH (*N* = 32)	*p* value
Age	53.5 (26.0–70.0)	49.0 (22.0–74.0)	0.247	53.5 (26.0–70.0)	52.0 (27.0–74.0)	0.633
Sex (M:F)	30:6	61:6	0.400	28:4	28:4	1.000
BMI (kg/m^2^)	23.0 (17.6–29.3)	22.0 (16.4–27.7)	0.091	22.8 (17.6–29.3)	22.2 (18.2–27.7)	0.788
HBV-DNA positivity	20 (55.6)	35 (52.2)	0.748	18 (56.3)	15 (46.9)	0.453
ASA grade			0.099			0.355
I	1 (2.8)	0 (0)		1 (3.1)	0 (0)	
II	29 (80.6)	46 (68.7)		25 (78.1)	23 (71.9)	
III	6 (16.7)	21 (31.3)		6 (18.8)	9 (28.1)	
TB (µmol/L)	12.8 (4.8–30.4)	14.5 (5.7–36.6)	0.145	12.8 (6.8–30.4)	12.5 (5.7–34.7)	0.851
ALB (g/L)	42.3 (35.5–48.0)	40.6 (25.3–46.8)	0.027	42.3 (35.5–48.0)	42.1 (32.5–46.8)	0.619
AST (IU/L)	39.0 (20.0–206.0)	46.0 (23.0–308.0)	0.054	36.5 (20.0–206.0)	43.0 (24.0–259.0)	0.131
ALT (IU/L)	39.0 (14.0–385.0)	49.0 (18.0–408.0)	0.066	39.0 (14.0–385.0)	48.0 (18.0–265.0)	0.114
PLT (10^9^/L)	127.0 (51.0–260.0)	99.0 (32.0–295.0)	0.013	138.0 (51.0–204.0)	105.0 (38.0–295.0)	0.405
WBC (10^9^/L)	5.2 (2.5–8.2)	5.4 (2.1–11.1)	0.288	5.2 (2.5–8.1)	5.2 (2.4–9.2)	0.555
AFP level (ng/mL)	10.9 (1.5–1210.0)	90.6 (2.2–1210.0)	0.054	10.9 (1.5–1210.0)	55.9 (2.4–1210.0)	0.105
ICGR-15 min (%)	4.8 (1.2–9.8)	4.9 (1.5-9.8)	0.981	4.8 (1.2–9.8)	4.9 (1.5–9.8)	0.672
Previous abdominal surgery	7 (19.4)	11 (16.4)	0.700	6 (18.8)	8 (25.0)	0.545
Comorbidities			0.138			0.449
Diabetes mellitus	1 (2.8)	11 (16.4)		1 (3.1)	4 (12.5)	
Hypertension	3 (8.3)	5 (7.5)		2 (6.3)	2 (6.3)	
COPD	3 (8.3)	3 (4.5)		2 (6.3)	3 (9.4)	
Tumor size (cm)	4.3 (1.0–10.0)	6.0 (1.5–10.0)	0.036	4.0 (1.0–10.0)	6.2 (1.5–10.0)	0.163
Tumor number (single)	33 (91.7)	61 (91.0)	1.000	29 (90.6)	29 (90.6)	1.000
Posterosuperior segments	15 (41.7)	33 (49.3)	0.462	11 (34.4)	14 (43.8)	0.442
R0 resection	35 (97.2)	63 (94.0)	0.812	31 (96.9)	30 (93.8)	1.000
Microvascular invasion	13 (36.1)	24 (35.8)	0.977	11 (34.4)	12 (37.5)	0.794
Capsular invasion	22 (61.1)	34 (50.7)	0.314	18 (56.3)	19 (59.4)	0.800
Satellites present	3 (8.3)	11 (16.4)	0.401	3 (9.4)	7 (21.9)	0.168
Poor differentiation	17 (47.2)	22 (32.8)	0.151	15 (46.9)	10 (31.3)	0.200

*BMI* body mass index, *HBV*-*DNA* positivity was defined as >1000 IU/mL, *ASA* American Society of Anesthesiologists, *TB* total bilirubin, *ALB* albumin, *AST* aspartate transaminase, *ALT* alanine transaminase, *PLT* platelet, *WBC* white blood cell, *AFP* alpha fetoprotein, *ICGR*-*15* indocyanine green retention rate at 15 min, *COPD* chronic obstructive pulmonary disease

### Surgical data and postoperative outcomes

Surgical data and postoperative outcomes of the two groups were shown in Table 2. Significant differences were observed in operative time (median, 255 vs. 210 min, *p* < 0.001; 255 vs. 200 min, *p* < 0.001, respectively) and Pringle time (median, 50 vs. 30 min, *p* < 0.001; 50 vs. 30 min, *p* < 0.001, respectively) both before and after PSM. The blood loss in LMH group was less than that of OMH group (median, 300 vs. 400 mL, *p* = 0.016), even though no significant difference was found after PSM (median, 300 vs. 325 mL, *p* = 0.186). The rate of blood transfusion did not differ both before and after PSM.

**Table 2:** Intraoperative data and postoperative outcomes before and after propensity score matching

Variables	Before matching			After matching		
	LMH (*N* = 36)	OMH (*N* = 67)	*p* value	LMH (*N* = 32)	OMH (*N* = 32)	*p* value
Surgical data						
Operative time (min)	255 (110–500)	210 (155–255)	<0.001	255 (110–460)	200 (165–245)	<0.001
Blood loss (mL)	300 (50–1000)	400 (50–2000)	0.016	300 (50–1000)	325 (50–2000)	0.186
Pringle time (min)	50 (20–150)	30 (15–80)	<0.001	50 (20–100)	30 (15–60)	<0.001
Blood transfusion	2 (5.6)	13 (19.4)	0.057	1 (3.1)	3 (9.4)	0.606
Postoperative outcomes						
Hospital stay (days)	8 (4–22)	9 (5–23)	0.017	7.5 (4–22)	9 (5–17)	0.060
Overall complications	11 (30.6)	36 (53.7)	0.024	10 (31.3)	12 (37.5)	0.599
Major complications	0 (0)	12 (17.9)	0.017	0 (0)	5 (15.6)	0.062
Liver-specific complications	10 (27.8)	36 (53.7)	0.012	9 (28.1)	11 (34.4)	0.590
Liver failure	8 (22.2)	14 (20.9)	0.876	7 (21.9)	5 (15.6)	0.522
Hemorrhage	1 (2.8)	3 (4.5)	1.000	1 (3.1)	0 (0)	1.000
Ascites	4 (11.1)	25 (37.3)	0.005	3 (9.4)	10 (31.3)	0.030
Biliary leakage	0 (0)	6 (9.0)	0.159	0 (0)	1 (3.1)	1.000
General complication	2 (5.6)	13 (19.4)	0.057	2 (6.3)	6 (18.8)	0.257
Respiratory infection	1 (2.8)	9 (13.4)	0.164	1 (3.1)	4 (12.5)	0.352
Wound infection	1 (2.8)	6 (9.0)	0.437	1 (3.1)	2 (6.3)	1.000
Pleural effusion	1 (2.8)	9 (13.4)	0.164	1 (3.1)	5 (15.6)	0.198
Cost (dollars)	7497.1 (4565.1–11,252.2)	5471.9 (2095.8–9966.8)	<0.001	7311.0 (4565.1–11,232.3)	5496.6 (2815.4–9966.8)	<0.001
90-day mortality	0 (0)	1 (1.5)	1.000	0 (0)	1 (3.1)	1.000

Postoperative outcomes showed that hospital stay was shorter in the LMH group than in the OMH group (median, 8 vs. 9 days, *p* = 0.017), even though there was no significant difference after PSM (median, 7.5 vs. 9 days, *p* = 0.060). Before PSM, the rates of overall complications (30.6 vs. 53.7%, *p* = 0.024), major complications (0 vs. 17.9%, *p* = 0.017), and liver-specific complications (27.8 vs. 53.7%, *p* = 0.012) were statistically different between two groups. However, the overall complication rate (31.3 vs. 37.5%, *p* = 0.599) as well as major complication rate (0 vs. 15.6%, *p* = 0.062) were comparable after PSM. Significant difference was noted in rate of liver-specific complications after PSM according to ascites (9.4 vs. 31.3%,*p* = 0.030) between the two groups. The rates of general complications in terms of respiratory infection, wound infection and pleural effusion did not differ between the groups. The overall cost of hospitalization (median, 7311.0 vs. 5496.6 dollars, *p* < 0.001) was much expensive in the LMH group than that of OMH group. The 90-day mortality was comparable between the two groups.

### Survival

**Fig. 3:**
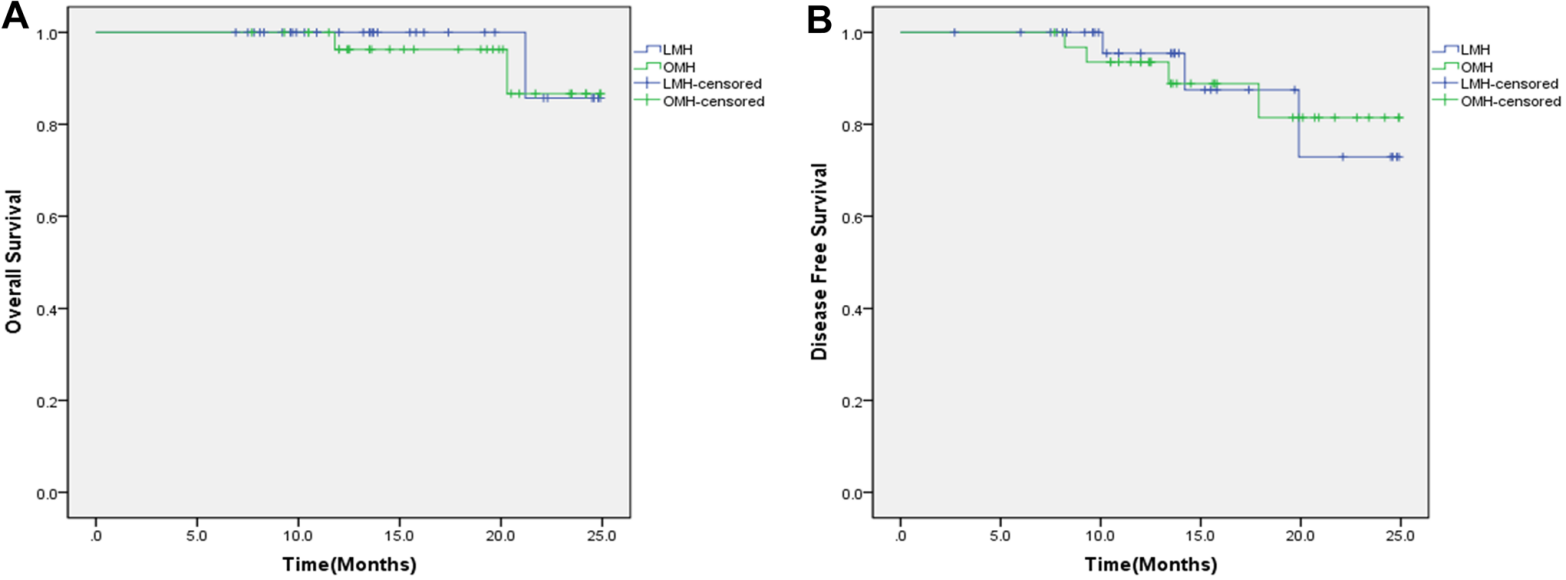
**A** Overall survival rates between the two groups (*p* = 0.694); **B** disease-free survival rates between the two groups (*p* = 0.990)

The median follow-up time was 13.8 (5.7–24.9) months. The oncological outcomes between the two groups did not differ with regard to OS (*p* = 0.694) and DFS (*p* = 0.990) after PSM. The 1- and 2-year OS rates were 100 and 85.7%, respectively, in the LMH group and 96.3 and 86.7%, respectively, in the OMH group (Fig. 3A). The 1- and 2-year DFS rates were 95.5 and 72.9%, respectively, in the LMH group and 93.5 and 81.5%, respectively, in the OMH group (Fig. 3B).

## Discussion

Hepatectomy remains the first-line treatment option for early- and intermediate-stage HCC due to the extremely limited number of donor supply worldwide [2]. Although recent advances in laparoscopic instruments and technique have greatly promoted the development of LLR, this procedure is still associated with challenge and technological complexity, especially for cirrhotic patients requiring major liver resection [3–5, 14].

The first laparoscopic liver surgery performed in our center was local resection for lesion positioned in segment III in 2009, and since then an increasing number of laparoscopic minor hepatectomy was introduced, mainly for left lateral section ectomy and wedged resection. Up to 2015, more than 40 cases of LLR were performed including both benign and malignant tumors and the first case of laparoscopic right hemihepatectomy was successfully operated, making the indications for LLR more expanded in our center. As it has been proved that this relatively novel technique is associated with a steep learning curve about 45–75 cases, the current study included patients underwent LLR only after 2015 to limit the influence of learning curve as less as possible [26–28]. As far as we are concerned, for safely starting the LLR, a comprehensive understanding of liver anatomy, basically learning of laparoscopic techniques in other abdominal surgery, knowledge of the merits and faults of different energy devices, and extensive open liver resection experiences are of great importance. Mastering each of these factors is a challenging task, and the requirement for combining all of them in LLR will no doubt contribute to the steep learning curve of LLR. Moreover, such a steep learning curve could be more obvious in LMH, which often requiring more laparoscopic minor liver resection and open major resection experiences. In our center, the comparison of LMH between 2015 and 2016 showed that even though patients in 2015 were associated with longer operative time and more blood loss compared with those in 2016, the results did not reach significant differences, indicating that the effect of learning curve was mitigated.

In the present study, our results suggested that the blood loss and the intraoperative transfusion requirement in the LMH group tended to be less than the OMH group, even though the comparable result was noted after PSM. Control of bleeding is of great concern in the field of liver surgery, particularly for LMH. Besides the meticulous dissection and maintaining low level of the central venous pressure during the liver parenchyma dissection, we routinely applied intermittent Pringle maneuver to control surgical blood loss. Differ from the previous series reported, the Pringle maneuver was performed occasionally in the event of major bleeding [29, 30], we adopted this technique as routine use because parenchyma transection in the setting of elevated portal pressure and impaired coagulation in cirrhotic patients can be extremely difficult. Under the magnified vision during laparoscopic hepatectomy, a clean surgical field with less blood loss is associated with shorter operative duration. Usually, the use of CUSA or harmonic scalpel in the setting of bleeding can easily stain the laparoscope, which may in turn affect the fluency of surgery and prolong the operative time. Therefore, the routine use of Pringle maneuver can sometimes be advantageous and help surgeons to control bleeding.

The relatively longer operative duration in the LMH group than that of OMH group could be attributed to the wide application of Pringle maneuver during parenchyma transection. What’s more, the effect of learning curve may still serve as an important role for the slow-gestating of LLR in our center compared with more than one thousand open liver resection experience over the past few years.

With respect to postoperative morbidity, the LMH group tended to be more superior than OMH group even though there were no statistical differences in the rates of overall complications, major complications, and liver-specific complications after PSM. Considering the liver-specific complications, the high rates of posthepatectomy liver failure and ascites were encountered after major hepatectomy which in line with the reported literatures [14, 29]. In current study, the proportion of liver failure did not differ between the two groups, while the incidence of ascites was significantly less in the LMH group than in the OMH group both before and after PSM, which also in compliance with the previous studies [14, 31]. Instead of making the large subcostal incision, the LMH simply put four or five trocars in the upper quadrant of abdomen, resulting in minimizing the destruction of the collateral circulation of the abdominal wall and lymphatic flow of the diaphragm in the setting of liver cirrhosis hence decreased the incidence of postoperative ascites.

It has been reported that LLR decreased the rates of infectious complications in the postoperative course [32]. In the current study, the OMH group seems more vulnerable to such complications, although there was no statistically significant difference in the incidence of general complications according to respiratory infection, wound infection and pleural effusion between the two groups before and after PSM. The hospital stay and 90-day mortality were comparable between the groups, while the cost of laparoscopic surgery was much more expensive compared with traditional open liver resection, which can be explained by the relatively new developed devices applied in the LMH such as LigaSure, high-definition laparoscope, and endoscopic stapler.

Based on the current study, the comparable oncological outcomes between the LMH group and the traditional open surgery group were observed after PSM. As it has been proved that the prognosis of HCC patients could be extremely influenced by the biological behavior of the hepatic tumors [33, 34], the results of pathological characteristics between the compared groups showed no statistically significant differences with respect to the R0 resection, microvascular invasion, capsular invasion, satellites present and poor differentiation, which certified the reliability of oncological outcome analysis of the current study.

There are some limitations in the present study. The small sample size, retrospective nature, and absence of randomization may limit the strength and validity of the results. However, given the fact that the LMH for cirrhotic patients is associated with novelty and unpredictable risk, the current study enrolled the relatively large number of cases compared with a lack of published data regarding this special cohort of patients. Although a randomized controlled trial may provide the most robust evidence for clinical study, it is sometimes extremely difficult to carry out as there is no accurate evaluation for the severity of liver fibrosis preoperatively, and it is unlikely to recruit patients when the two techniques are associated with obviously different cosmetic effect. To overcome the selection bias arising from lack of randomization, we performed the PSM analysis which is deemed as the most effective method to balance the covariates and thus reducing bias in the retrospective studies. Despite this, it could lead to the reduction of the original small number of cases and the inevitable loss of information. Further studies with large sample size are definitely warranted.

In conclusion, the current study demonstrated that LMH for HCC patients with liver cirrhosis showed comparable results in terms of postoperative morbidity and oncological outcomes compared with traditional open procedure. LMH may serve as a safe and feasible alternative for selected HCC patients with cirrhosis.
